# Myocardial extracellular volume imaging by CMR quantitatively characterizes myocardial infarction and subclinical myocardial fibrosis

**DOI:** 10.1186/1532-429X-13-S1-P148

**Published:** 2011-02-02

**Authors:** Martin Ugander, Abiola J Oki, Li-Yueh Hsu, Peter Kellman, Andreas Greiser, Marcus Y Chen, W Patricia Bandettini, Anthony H Aletras, Andrew E Arai

**Affiliations:** 1 National Institutes of Health, Bethesda, MD, USA; 2 Siemens AG Healthcare Sector, Erlangen, Germany

## Introduction

Imaging of fibrosis by cardiovascular magnetic resonance (CMR) is performed using late gadolinium enhancement (LGE). However, LGE imaging only visualizes relative differences between “normal” myocardium and fibrosis or infarction. Recent developments in T1 mapping have made it practical to quantitatively image the extracellular volume fraction (ECV).

## Purpose

To quantify ECV in patients. We hypothesized that 1) ECV imaging could quantitatively differentiate LGE lesions from normal myocardium, 2) ECV of non-infarcted myocardium would vary with age, and 3) ECV would vary in myocardium remote from infarction.

## Methods

Patients (n=126) were imaged at 1.5T (Siemens) with a Modified Look-Locker Inversion-recovery (MOLLI) sequence before and approximately 15 minutes after a 0.15 mmol/kg bolus of Gd-DTPA. T1 and R1 pixel maps were generated. DeltaR1 maps (R1after - R1before contrast) were divided by the DeltaR1 value of the LV blood pool and multiplied by [1- hematocrit], yielding a quantitative pixel map of the ECV ranging from 0-100%. LGE images were acquired for the entire left ventricle.

## Results

**Figure 1:**
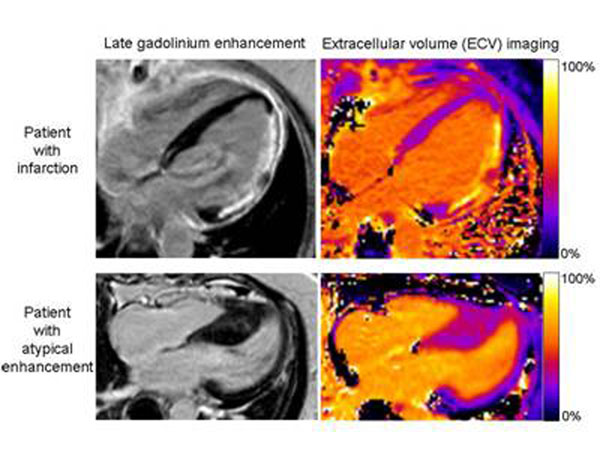
LGE and ECV imaging in two representative patients.

In patients with no clinically detected focal abnormalities by LGE (n=60, 31 male, mean±SD age 50±17 years), the ECV of the myocardium was (mean±SD) 26±3%. In patients with infarction by LGE (n=36, 31 male, age 58±12 years), the ECV of remote myocardium was 27±3% (p=ns vs patients with normal LGE) and the ECV of infarcted myocardium was 51±8% (p<0.001). In patients with atypical enhancement by LGE (n=30, 23 male, age 54±11 years), the ECV of remote myocardium was 26±3% (p=ns vs patients with normal LGE) and the ECV of atypically enhanced myocardium was 37±7% (p<0.001). In patients with infarction, the ECV of remote myocardium increased as left ventricular ejection fraction decreased (r=-0.49, p=0.002). In infarcted and non-infarcted patients (n=96), as well as both subsets, the ECV of “normal” myocardium increased with age (r=0.27, p=0.004). ECV of “normal” myocardium did not increase with age in the atypical enhancement group. Figure 1

## Conclusions

ECV imaging is a novel tool for quantitative characterization of both focal and diffuse abnormalities in the myocardium, beyond what is assessable by LGE. Infarctions varied considerably in ECV, but displayed no overlap with ECV of “normal” myocardium. Atypical enhancement showed a larger variability and some overlap with “normal” myocardium. ECV in “normal” myocardium is consistent with age related subclinical fibrosis. ECV imaging also detects a subtle abnormality in myocardium remote from infarction which may represent adverse post-infarct remodeling.

